# The spatial dynamics of immune response upon virus infection through hybrid dynamical computational model

**DOI:** 10.3389/fimmu.2023.1257953

**Published:** 2023-11-06

**Authors:** Yanan Cai, Zhongrui Zhao, Changjing Zhuge

**Affiliations:** Department of Mathematics, Faculty of Science, Beijing University of Technology, Beijing, China

**Keywords:** SARS-CoV-2, immune response, cellular automata, hybrid model, virus diffusion, antibody

## Abstract

**Introduction:**

The immune responses play important roles in the course of disease initiation and progression upon virus infection such as SARS-CoV-2. As the tissues consist of spatial structures, the spatial dynamics of immune responses upon viral infection are essential to the outcome of infection.

**Methods:**

A hybrid computational model based on cellular automata coupled with partial differential equations is developed to simulate the spatial patterns and dynamics of the immune responses of tissue upon virus infection with several different immune movement modes.

**Results:**

Various patterns of the distribution of virus particles under different immune strengths and movement modes of immune cells are obtained through the computational models. The results also reveal that the directed immune cell wandering model has a better immunization effect. Several other characteristics, such as the peak level of virus density and onset time and the onset of the diseases, are also checked with different immune and physiological conditions, for example, different immune clearance strengths, and different cell-to-cell transmission rates. Furthermore, by the Lasso analysis, it is identified that the three main parameters had the most impact on the rate of onset time of disease. It is also shown that the cell-to-cell transmission rate has a significant effect and is more important for controlling the diseases than those for the cell-free virus given that the faster cell-to-cell transmission than cell-free transmission the rate of virus release is low.

**Discussion:**

Our model simulates the process of viral and immune response interactions in the alveola repithelial tissues of infected individuals, providing insights into the viral propagation of viruses in two dimensions as well as the influence of immune response patterns and key factors on the course of infection.

## 1 Introduction

The consequences of infecting SARS-CoV-2 relies on host-virus interaction, which includes a complicated processes such as replication and invasion of virus, and the triggering of immune response [Wadman et al. (1)]. Mathematical modeling of viral infection within the host is commonly employed as a research approach [Perelson et al. (2); Nowak et al. (3); Elaiw and Agha (4)]. These studies typically build upon classical viral kinetic models, extending their applications to determine the basic reproduction number of SARS-CoV-2 during host growth and analyze the dynamic evolution of viral density and different cell populations [Elaiw and Almuallem (5); Li et al. (6)]. The immune response plays a crucial role in controlling the long-term spread of the virus, but it undergoes decay over time, and reinfection can occur in the human body. Therefore, it is of utmost importance to comprehend the mechanisms governing immune control and the dynamics of immune memory in response to the virus. This understanding is vital for studying the propagation of SARS-CoV-2 within the host and conducting experiments to explore its interactions with the immune system [Du and Yuan (7); Ghosh (8); Wang et al. (9); Mondal et al. (10)].

Based on the evolution, development, and immune effector mechanisms of the individual immune system, immunity is typically divided into innate immunity and adaptive immunity [Cao and He (11)]. The innate immune system recruits immune cells to phagocytose and eliminate infected cells by recognizing pathogens and inducing the production of pro-inflammatory factors and chemokines. It plays a crucial role in clearing viruses during the early and middle stages of the immune response [Chowdhury et al. (12)]. However, it has been shown that unregulated expression and signaling of interferons and excessive production of pro-inflammatory cytokines can exacerbate the disease in infected individuals if not properly regulated [Lowery et al. (13)].

Simultaneously, the initiation of innate immunity triggers adaptive immunity response involving both humoral and cellular immunity. For extracellular viruses, antibodies specifically recognize the antigenic components of the virus, neutralizing them and preventing the invasion of healthy cells. In the case of intracellular viruses, where specific antibodies cannot directly enter cells and bind to them, T cells are required to mediate cellular immunity. This cellular immunity involves directly lysing and destroying infected virus cells for clearance or enhancing the immune response of T cells, macrophages, and B cells to the virus through the lymphokines they secrete, ultimately removing the virus from the cells [Hernandez-Vargas and Velasco-Hernandez (14)].

Therefore, in contrast to previous studies focusing on innate or adaptive immunity components, this paper places greater emphasis on the role of humoral and cellular immunity to the virus during the middle and late stages of infection. These aspects are incorporated as influencing factors in the hybrid model we constructed to simulate the spread and dissemination of novel coronaviruses in humans.

Viral transmission within the host exhibits complex spatiotemporal dynamics and evolutionary behavior [Cilfone et al. (15); Juhasz et al. (16)]. Some studies primarily focus on specific time scales and lack the necessary cross-scale communication to capture biochemical reaction processes adequately [Baris Hancioglu and Swigon (17); Howat et al. (18); Graw and Perelson (19); Kyrychko et al. (20)]. Conversely, hybrid multiscale modeling has been extensively utilized in tumor growth dynamics and influenza virus kinetics, with a particular emphasis on the cell spreading process and drug inhibition [Bravo et al. (21); Anderson (22); Gerlee and Anderson (23); Rejniak and Anderson (24)].

To enhance the exchange of information between the spatial scale of immune response and viral dynamics, we developed a hybrid kinetic model combining partial differential equation theory with Metacellular Automata, a tool capable of capturing spatiotemporal effects. This model enables us to analyze the impact of different forms of immunity on the diffusion pattern, peak virus levels, and infection cycles of novel coronaviruses [Peter et al. (25); Macal and North (26); Arduin et al. (27); Bar-On et al. (28); Carcaterra and Caruso (29); Mason (30)].

Furthermore, the relationship between SARS-CoV-2 viral density and the risk of disease progression remains poorly defined [Fajnzylber et al. (31); Zou et al. (32)]. Exploring this relationship is crucial for stratifying the severity of COVID-19 in infected patients. In contrast to available clinical data, we attempted to establish an onset baseline by determining the percentage of infection and simulating the relationship between the time of onset and some critical parameters in our model. These parameters can be obtained via Lasso analysis and OSL regression analysis. Thereore, we can further analyze the spreading process of the virus under different modes of immunity as well as the susceptibility factors affecting the pathogenic nodes.

## 2 Materials and methods

### 2.1 Model description

Upon infection, the virus enters the throat through the nasal and oral cavities. This is a complicated spatiotemporal process involving many cells distributed along respiratory tract and alveoli, it is necessary to simulate the processes by taking the spatial effects into consideration. Although the real tissue is organized as a three dimensions of cell architectures, the free virus to initialize the processes of the infection are distributed on the surface of epithelium which is approximately a thin 2-dimension layer of cells. And furthermore, cells show the phenotype of an epithelial monolayer and exhibit lung-progenitor-like expression patterns under 2-dimensional culture *in vitro* experiments as well as many computational models show consistence results of 2-dimensional models, [Tran et al. (33); Heydemann et al. (34); Yang et al. (35); Wessler et al. (36)]. So we choose the 2-dimensional model in the current study.

**Figure 1:**
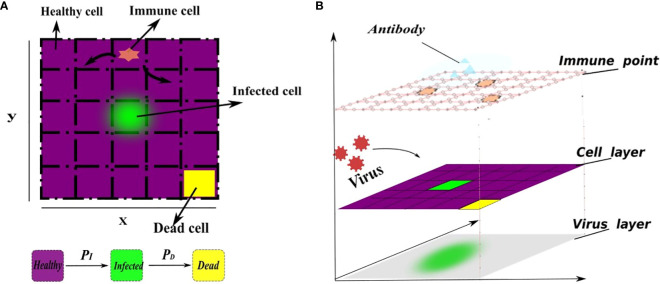
A schematic representation of the cell transformation. **(A)** In the flat view representation, **(B)** The structure of metacellular automata. Every grid square is assigned three state values, namely, healthy (0), infected (1), or dead (2), which are represented by the function *S_i,j_* (*t*). The healthy grid square is indicated by the color purple, the infected grid square is represented by the color green, and the dead cells are depicted in yellow. Specifically, the infected grid square (green) release the virus, enabling its spread throughout the system. Initially, the virus density across the entire region is 0, indicating no viral presence. A small number of cells are randomly selected to be in an infected state, while the remaining cells maintain a healthy state. Furthermore, each grid within the region Ω has the capacity to accommodate at least one immune cel that will release antibodies to clear virus, allowing immune cells to freely move to any position within the grid. The virus is released from infected cells and spreads to the surrounding area. The transition of each square’s state depends on several factors, including the current grid’s viral density, the state of neighboring grids, and the presence of immune grids. Higher grid’s viral density increase the susceptibility of healthy grid squares to infection. Additionally, the presence of immune grids enhances the death rate of infected grid squares. This corresponds to the direct killing of infected cells by immune cells. The infection probability (*P_I_*) depends on the rate of cell-to-cell transmission and free-virus mediated infection. Similarly, the mortality probability (*P_D_*) depends on natural mortality of infected cell and the number of immune cells. This relationship is defined as a linear combination for simplicity. In addition, the cellular neighborhood is determined using Moore’s neighborhood, which encompasses the 8 adjacent cells.

The model consists of four layers of cellular automata, each of which represents the immune cells, the epithelial cells, the distribution of virus and the distribution of antibody (**Figure 1**). For the layers of immune cells and epithelial cells. We define a planar bounded region Ω to represent the infection region of the alveolar epithelial cell tissue in the context of the novel coronavirus.The region Ω is partitioned into 250 × 250 grid using two sets of parallel lines, where the coordinates *x* = *i* and *y* = *j* (*i*, *j* =1, 2,…, 250) represent the indices of the grid squares. Each grid square in the partition corresponds to one epithelial cell, resulting in a total of 62,500 epithelial cells. This chosen value falls within a reasonable range for the simulation, allowing for a comprehensive representation of the cellular landscape in the tissue [Juhasz et al. (16); Marino and Kirschner (37)]. Each grid square is denoted as Ω*_i,j_*, where *i* represents the row index and *j* represents the column index. All pictures of model description can be skeched by **Figure 1**.

**Figure 2:**
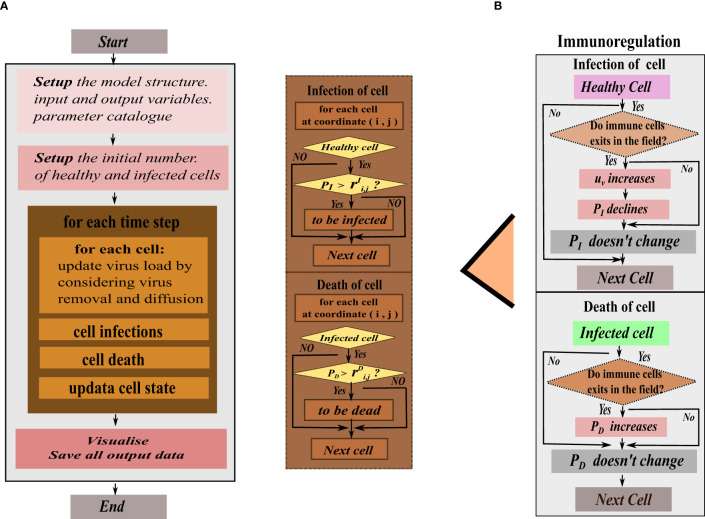
The program flow diagram of the hybrid model’s simulation. **(A)** In the flat view representation, **(B)** The structure of metacellular automata.

In reality, grid square’s state transition is a highly random process, so in our model, the meta-automaton incorporates two parameters: the infection rate (*P_I_*) for healthy grid square and the mortality rate (*P_D_*) for infected cells. During each time unit, the meta-automaton iterates over all grids. For a healthy grid square, the cell automaton generates a random number between 0 and 1, denoted as $r_{i,j}^{I}$ . If the infection rate *P_I_* of that healthy cell is greater than $r_{i,j}^{I}$ , the healthy grid will turn into an infected grid. Otherwise, the grid state remains unchanged. Similarly, another random number $r_{i,j}^{D}$ is generated between 0 and 1 for an infected grid. The mortality rate *P_D_* is compared with this random number, and if *P_D_* is greater than $r_{i,j}^{D}$ , the infected cell will turn into a dead grid. To illustrate this dynamical change, we combine the given model operation process of cellular automata. At the initial moment, set the input and output variables of the model and the parameters in the cellular automata. Then, the meta-cellular automaton performs the following steps during each time unit. Firstly, calculate viral density in each grid square using a specific equation, considering virus proliferation, removal and diffusion. Among them, the clearance of the virus is regulated by immune cells, antibodies, and innate proliferation rate. In this step, according to the previously set immune mode, the cellular automata determines if the virus is affected only by intrinsic clearance. If there is no immune response, the program follows the rule of **Figure 2A**, otherwise follow the **Figure 2B** on the right. Secondly, record the virus and antibody matrix at the current moment. Thirdly, perform grids’ state transitions based on the defined rules. Finally, update and record the current grid state matrix. This process is repeated until all grids are traversed and their state no longer change.

The infection rate *P_I_* in healthy cells is dependent on the current intracellular viral density and the infection rate between cells, which means that cellular infection is jointly mediated by free viruses and direct cell-cell transmission. Cell-to-cell transmission manifests as viral transmission between adjacent cells. Therefore, cellular automata are limited to the adjacent range of 8 grid squares when simulating infected cell could tran infecting surrounding healthy grids. The mortality rate *P_D_* depends on natural mortality of infected cell and the clearance of immune cells. However, in the immunity-free mode, only the natural death rate constant is considered. When the immune system responds, immune cells release antibodies to clear the virus, and the rate (*u_v_*) of virus removal increases. The immune cells directly kill infected cells, (*P_D_*) increases, and the probability of healthy cells being infected (*P_I_*) decreases.

Regarding infected cells, the viral source term *ρ*(*t,x,y*) does not adhere to a specific form, and any reasonably smooth function *ρ*_0_(*t,x,y*) is permissible, considering our limited knowledge of viral outbreak characteristics such as form, duration, and size. To simplify the situation, we assume that infected cells exhibit a constant rate of virus production, as supported by relevant studies. Therefore, *ρ*_0_ is treated as a constant. Furthermore, *ρ*_0_ must satisfy the following conditions.

- When a healthy cell at coordinates (*i*,*j*) becomes infected, it initiates the secretion of viral particles. As a result, *ρ*_0_ assumes a positive value for at least some subset of Ω*_i,j_* at a certain point following the infection.
- To ensure the stability of the system, the viral source term *ρ*_0_(*t,x,y*) must be defined in a manner that guarantees the fitness of the system. Specifically, *ρ*_0_(*t,x,y*) should exhibit Holder¨ continuity with respect to a parameter *α* in the interval (0, 1) to ensure the global existence of the solution.

The immune response elicited by viruses is a complex process influenced by various regulatory forms and target objects, along with the time required for immune cells to recognize viruses and present antigens. Consequently, virus clearance involves a sophisticated mechanism. In the equation, the coefficient *u_v_* in the second term is not a simple constant due to the time lag. *u_v_* represents the rate of virus removal, indicating how efficiently the immune system or other clearance mechanisms remove the virus from the cell, and it is mainly regulated by intrinsic clearance, antibody levels and immune cells, which is a linear combination for simplified model. Considering that immune cells are able to recognize infected cells and activate a series of signals to recruit more immune cells, *u_v_* and the number of immune cells surrounding the infected cells are linearly related, as well as depending on the location of the infected cells. In fact, humoral immunity is established the antibodies will be present everywhere, so *u_v_* is larger than zero in any place. We consider antibody levels that are also described by a diffusion equation. It mainly includes a diffusion item, a proliferation item and a natural attenuation item. The proliferative item is regulated by virus density, the number of immune cells and infected cells. This regulation follows the form of the Hill function to indicate the positive feedback effect of inducing antibody production. In addition, adaptive immunity also includes cellular immunity, i.e., T lymphocytes can directly recognize and kill infected cells, increasing the mortality of infected cells, which is not directly presented in Eq.(2). Finally, we considered that the infection process of infected cells involves two factors, mediated by free virus and neighboring cell-to-cell transmission. At each moment, the viral density at different locations is affected by the spread of the virus at the previous moment, and the probability that a healthy cell at the current location is transformed into an infected cell is correlated with the viral density [Clausen et al. (38)], which may be related to the release of free virus from infected cells in non-neighboring areas at the previous moment. For healthy cells in which infected cells are present in the neighborhood, viruses can spread directly through cell-to-cell mediated transmission. Other studies have shown that the rate of cell-to-cell transmission is about 10 times that of virus-mediated infection. {Zeng et al. (39); Kruglova et al. (40)]

### 2.2 The diffusion of cell-free virus and antibody

As the processes of infection depend on the contact between healthy cells and viral particles or infected cells, the infection rate *P_I_* of healthy cells is assumed to be dependent on both of the cell-to-cell transmission and cell-free viral load. The viral density in the cell with coordinates (*x,y*) at moment *t* is denoted by *V* (*t,x,y*). We assume that the cell-free virus particles are diffusing across the space among cells, so the diffusion processes can be modeled by the following diffusion equation (1).


(1) $$\frac{\partial V(t,x,y)}{\partial t}=D_{v}\Delta V,\;\left(x,y\right)\in \text{Ω}.$$

For simplicity, in this study, we simplify the equation (1) so as to consistent with the overall setting of cellular automata by assuming the density of cell-free virus are even in each grid square and the diffusion only occur between two adjacent squares. So the update formula of the cell-free virus due to diffusion can be written as (2).


(2) $$V\left(t+dt,x,y\right)=V\left(t,x,y\right)+D\left(t,x,y\right)dt.$$

where


$$D(t,x,y)=D_{v}\frac{V(t,x,+dx,y)+V(t,x-dx,y)+V(t,x,y+dy)+V(t,x,y-dy)-4V(t,x,y)}{dxdy},$$

*D*(*t,x,y*) is the discretion of the diffusion term and *D_v_* is the diffusion coefficients, and *dt*, *dx* and *dy* are the lengths of time step, *x*−grid squares and *y*−grid squares respectively. Moreover, as the infected cells produce and release free virus particles constantly, so it is necessary to add a source term (3) in the equation (2).


(3) $$\rho (t,x,y)=\{\begin{array}{ll} \rho_{0}, & \text{if the cell located at }(x,y)\text{ is infected },\\ 0, & \text{otherwise}. \end{array}$$

On the other hand, the degradation of free virus particles are attributed to three causes, the natural degradation of free virus particles, the clearance of virus by antibody and the removal of virus by immune cells. So the degradation terms can be modeled by the following formula.


(4) $$u_{v}=u_{v,0}+u_{v,antibody}+u_{v,immune}$$

where *u_v_*_0_ is the natural degradation of free virus particles, *u_v,antibody_* and *u_v,immune_* are the clearance of virus by antibody and the removal of virus by immune cells respectively, which is assumed to be proportional to the antibody level at the same grid square and the effective number of immune cells in the same grid square as well as in adjacent squares.

So, in summary, the dynamics of the cell-free virus particles is modeled as the following formula (5).


(5) $$V\left(t+dt,x,y\right)=V\left(t,x,y\right)+\left(D\left(t,x,y\right)-u_{v}V\left(t,x,y\right)+\rho \left(t,x,y\right)\right)dt.$$

The dynamical modelling of the levels of antibody is similar to those of virus, consisting of three term, the diffusion term, the degradation term and the source term. The modelling of the diffusion of antibody is the same as that of free virus particles where we also simplify the model by assuming that the level of antibody in each grid square is even and the diffusion occurs only between directly adjacent grid squares. The degradation of antibody is assumed to contain only the natural degradation. And, the source term for the antibody is not suggested by the generation of antibody in situ, but a simplification of the processes of recruiting antibody via any signaling of infected cells or virus or immune cells. The source term is proposed to be dependent on the level of free virus, the surrounding number of immune cells and surrounding number of infected cells through hill functions to simulate the saturation of the ability of recruiting antibody. So the source term of the antibody can be written as the following.


(6) $$\rho_{a}\left(t,x,y\right)=\rho_{a,v}\left(t,x,y\right)+\rho_{a,i}\left(t,x,y\right)+\rho_{a,c}\left(t,x,y\right),$$

where the *ρ_a,v_* (*t,x,y*),*ρ_a,i_* (*t,x,y*) and *ρ_a,c_* (*t,x,y*) denote the source terms attributed to free virus, immune cells sand infected cells as described above. And the three term can be expressed as $\rho_{a,s}=\rho_{a,x0}\frac{x^{2}}{K_{x}^{2}+x^{2}}$ . Here *x* = *v,i,c* as above, denoting the level of virus, the number of immune cells and the number infected cells. Here the “number” means that we counts the number of immune cells or infected cells in the full region. The *K_x_* are EC50’s of each hill function (**Table 1**).

**Table 1:** Model parameter Cases in different immune modes.

Number	Immune movement mode 1			Immune movement mode 2			Immune movement mode 3		
	Case I-1	Case I-2	Case I-3	Case II-1	Case II-2	Case II-3	Case III-1	Case III-2	Case III-3
*x*_1_	20	20	20	20	20	20	20	20	20
*x*_2_	43722	543	1473	21374	11428	352	29	170	30236
*x*_3_	0	0	0	0	0	0	0	0	0
*x*_4_	0	0	0	0	0	0	0	0	0
*x*_5_	0.0117	0.0039	0.0202	0.0012	0.002	0.0267	0.1012	0.0042	0.0775
*x*_6_	0.4782	0.0414	0.0064	0.0641	0.0475	0.0084	0.0237	0.0332	0.0051
*x*_7_	0.01	0.01	0.01	0.01	0.01	0.01	0.01	0.01	0.01
*x*_8_	0.0023	0.182	0.0422	0.0031	0.0047	0.0155	0.0074	0.114	0.0610
*x*_9_	0.037	0.0953	0.4766	0.0216	0.3198	0.0537	0.0099	1.0146	0.0242
*D_V_*	0.1	0.1	0.1	0.1	0.1	0.1	0.1	0.1	0.1
*ρ*_0_	2.5752	18,447	33.3715	0.7174	0.3836	35.4358	2.6161	37.1278	47.4464
*x*_10_	0.05	0.05	0.05	0.05	0.05	0.05	0.05	0.05	0.05
*x*_11_	0	0	0	0	0	0	0	0	0
*x*_12_	4.0636	8.5895	2.0489	2.061	24.5642	1.1404	4.7597	1.0194	6.3983
*x*_13_	0.1280	0.5081	0.9308	0.0802	3.1569	2.0528	0.3692	0.3334	4.3827
*x*_14_	0.011	0.0014	0.0223	0.0053	0.04343	0.1016	0.0026	0.002	0.0008
*x*_15_	0.1539	1.1436	0.6276	0.5086	0.0664	0.0818	0.1773	0.1301	0.1029
*x*_16_	0.5	0.5	0.5	0.5	0.5	0.5	0.5	0.5	0.5
*x*_17_	0.8	0.8	0.8	0.8	0.8	0.8	0.8	0.8	0.8
*x*_18_	0.1948	0.0961	0.1226	0.51	0.9187	0.0617	0.1439	0.1099	0.5052
*x*_19_	0.0633	0.07632	0.0035	0.0728	0.1255	0.0996	0.0607	0.0054	0.0636
*x*_20_	5	5	5	5	5	5	5	5	5
*x*_21_	0.3300	0.8218	0.6788	0.7020	0.0129	0.4324	0.4488	0.2763	0.0015
*x*_22_	0.7434	0.0194	0.5826	0.5268	0.6954	0.2203	0.4522	0.1823	0.6117
*x*_23_	0.07223	0.4026	0.0670	0.3733	0.3369	0.7952	0.6764	0.5395	0.5755

### 2.3 Estimation of parameters

The parameters involved in the model is shown in **Table 2**, we and carried out a large number of random simulations according to the scope of existing studies [Juhasz et al. (16); Baris Hancioglu and Swigon (17); Bocharov and Romanyukha (41)], from which several sets of simulation values were selected for subsequent research (**Table 1**).

**Table 2:** Parameters and initial conditions in the hybrid model.

Module	Interpretation of Parameters	Symbol in Table 1
Initial states	Initial number of infected cell	*x*_1_
	Initial number of immune cell	*x*_2_
	Initial virus level	*x*_3_
	Initial antibody level	*x*_4_
Epithelial module	Infection rate induced by virus	*x*_5_
	Infection rate induced by cell-to-cell transmission	*x*_6_
	Regeneration rate cell	*x*_7_
	Natural death rate of infected cell	*x*_8_
	Death rate of infected cell with immune	*x*_9_
Virus module	Diffusion rate of virus	*D_V_*
	Release rate of virus from infected cell	*ρ*_0_
	Natural clearance rate of virus	*x*_10_
	Burst size of virus	*x*_11_
	Clearance of virus induced by antibody	*x*_12_
	Clearance of virus induced by immune	*x*_13_
Immune module	Generation rate of immune cell	*x*_14_
	Degradation rate of immune cell	*x*_15_
	Random movement probability of immune cell	*x*_16_
	Directional movement probability of immune cell	*x*_17_
Antibody module	Generation rate of antibody	*x*_18_
	Degradation rate of antibody	*x*_19_
	Diffusion rate of antibody	*x*_20_
	EC50 generate antibody rate induced by immune cells	*x*_21_
	EC50 generate antibody rate induced by free virus	*x*_22_
	EC50 generate antibody rate induced by Infected cells	*x*_23_

The EC50 refer to a concentration or strength of a cell population or a concentration of components required to increase or reduce the measured response to half of the maximal level or concentration.

## 3 Results

This section presents the visualization of meta-cellular automata simulations aimed at elucidating the dynamics of virus spread. Specifically, we investigated the influence of immune cells, diffusion coefficients, and modes of immunosuppression by systematically manipulating the model parameters. Moreover, we established an infection value to define the onset and estimated the correlations between the time of onset and the aforementioned parameters. Through systematic parameter variations, our objective is to discern and analyze the factors governing the time for the onset.

### 3.1 Viral spread without immune modulation

**Figure 3:**
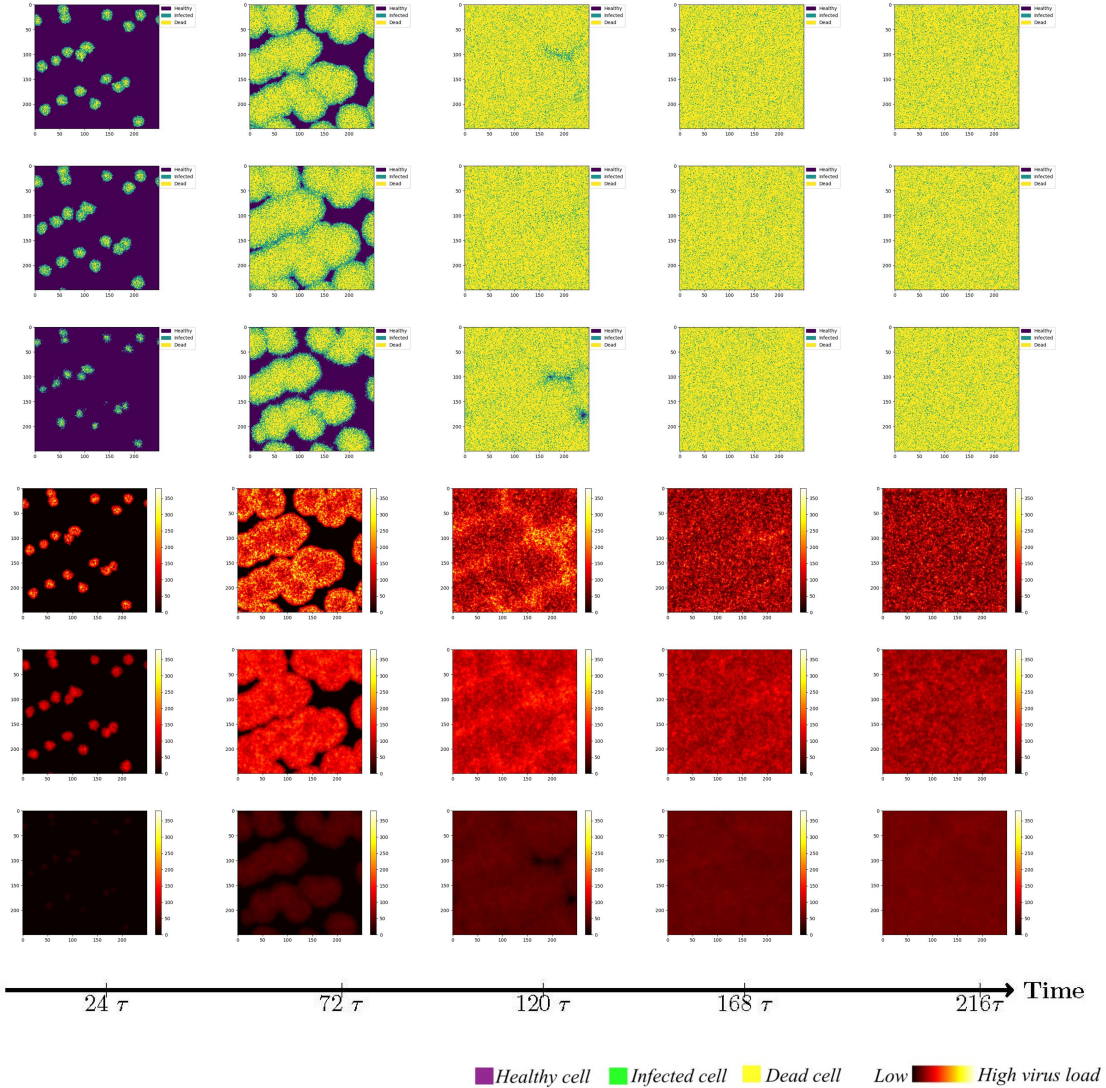
Cell state (Row 1, 2, 3) and virus diffusion (Row 4, 5, 6) at different time points with *D_V_* = 0.1 (Row 1 and 4), *D_V_* = 0.5 (Row 2,5) and *D_V_* = 5 (Row 3 and 6).

To investigate the impact of the immune system on the dissemination of viral infections within the human body, this section initially examines the simulation of virus spread in alveolar epithelial cells in the absence of immune regulation. Since other influencing factors are not considered, we only need to fix a set of parameter values to study for example. The simulation parameters are set in Case 1 of **Table 1**. At the initial time point, the system comprises a total of 62,500 cells, among which 20 are initially infected and randomly distributed across the area, while the remaining cells are healthy. The viral density is initialized as *x*_3 =_ 0. The propagation of the virus originates from the initially infected cell, and to capture the spreading process, cellular states are recorded at five-time points, with intervals of 48-time units, in order to account for the early stages of viral volume expansion and facilitate the observation of the spread dynamics. As depicted in **Figure 3**, the horizontal and vertical coordinates in each subplot are scaled to a range of 0 to 250, representing a spatial mapping of a human lung cell area with dimensions of 250 × 250 units. Each unit area corresponds to an individual lung cell, thus capturing the cellular landscape in a proportional manner. The first three rows show the visualization of the cell state under the diffusion coefficient (0.1, 0.5, 5). Lines 4-6 acts as the spread of the virus corresponding to these three cellular infections. The dynamics of viral spread exhibit a characteristic pattern of low dissemination. Initially, infected cells (represented by the color green) emerge individually, forming isolated clusters that resemble islands within the spatial distribution of the virus. These cellular communities exhibit well-defined boundaries and distinct lines of demarcation. It is worth noting that at this early stage, the infected areas remain disjointed and fragmented. Additionally, a closer examination reveals narrower regions of green coloration at the periphery of the cell communities, while the inner portions prominently display a predominance of dead cells (indicated by the color yellow). By observing the system at *t* = 120*τ*, the virus progressively spreads throughout almost the entire area, indicating a scenario where all cells would ultimately succumb to infection in the absence of immune system regulation. When the value of *D_V_* was increased from 0.1 to 0.5. As illustrated in rows 2 and row 4 of **Figure 3**, a distinct phenomenon emerges. Meanwhile, the number of infected cells is higher. From the perspective of virus spread scene, when *D_V_* = 0.1, the row 4 showed a higher concentration of virus at the edge of the infection area. When *D_V_* = 0.5, the row 5 indicated that the concentration distribution of virus was more uniform. However, the diffusion coefficient *D_V_* does not always have a positive effect on the spread of the virus. In the case of *D_V_* = 5 (Row 3 and 6), the virus spreads too quickly to concentrate on healthy cells, resulting in a smaller number of cells infected in a short period of time than in both cases. For *D_V_* = 0.1 or 0.5, the virus undergoes a rapid expansion followed by a slower progression, encompassing a complete life cycle of viral proliferation, peaking, and decay to an equilibrium state. Conversely, for the scenario with *D_V_* = 5, it takes longer for the viral density to reach its peak, suggesting that the diffusion coefficient *D_V_* exerts an influence on the spatial pattern of viral spread, the magnitude of peak viral density, and the duration of the infection cycle.

While maintaining the uniform spread of the virus, we found that at a low diffusion coefficient, the peak viral density was around *t*=120*τ* (Row 4 and 5). When *D_V_* = 5, it takes longer to peak. This shows that the diffusion coefficient of *D_V_* affects the spatial pattern of virus transmission and the time when the virus peaks. Through extensive simulations, it became evident that the spread pattern of the novel coronavirus, although initially displaying an island-like distribution, is characterized by a relatively weak intensity, susceptible to modulation by the diffusion coefficient, particularly when higher values are employed.

### 3.2 Viral spread in different immune modulation patterns

The immune system serves as the body’s defense mechanism, playing a crucial role in inhibiting the spread of viruses. Considering the response time and potency of the immune system, this section focuses on investigating the influence of adaptive immunity on virus propagation.

Adaptive immunity cells encompass both T lymphocytes and B lymphocytes. B lymphocytes exhibit antigen specificity, undergoing differentiation and antibody synthesis to eliminate antigens via humoral immunity. To represent the clearance effect of antibodies on the virus, a layer of antibody is added to the cellular automata. In cellular immunity, T lymphocytes recognize antigens, undergo activation and proliferation, and ultimately differentiate into effector T cells capable of specifically eliminating infected target cells through cytotoxic mechanisms. This is reflected by an augmented mortality rate of infected cells, denoted by the parameter *P_D_* within the meta cellular automata framework. To initiate the simulations, the diffusion coefficient is set to *D_V_* = 0.1. Additionally, 20 randomly chosen locations within Ω are designated as immune cell sites, while other conditions remain constant. While immune cells possess the ability to migrate, the complexities involved in this process are not considered in this study. Specifically, the physical hindrance imposed by other cells within the body on immune cell movement is not taken into account. In our simulation, immune cells are initially positioned at fixed locations and remain immobile throughout the experiment.

**Figure 4:**
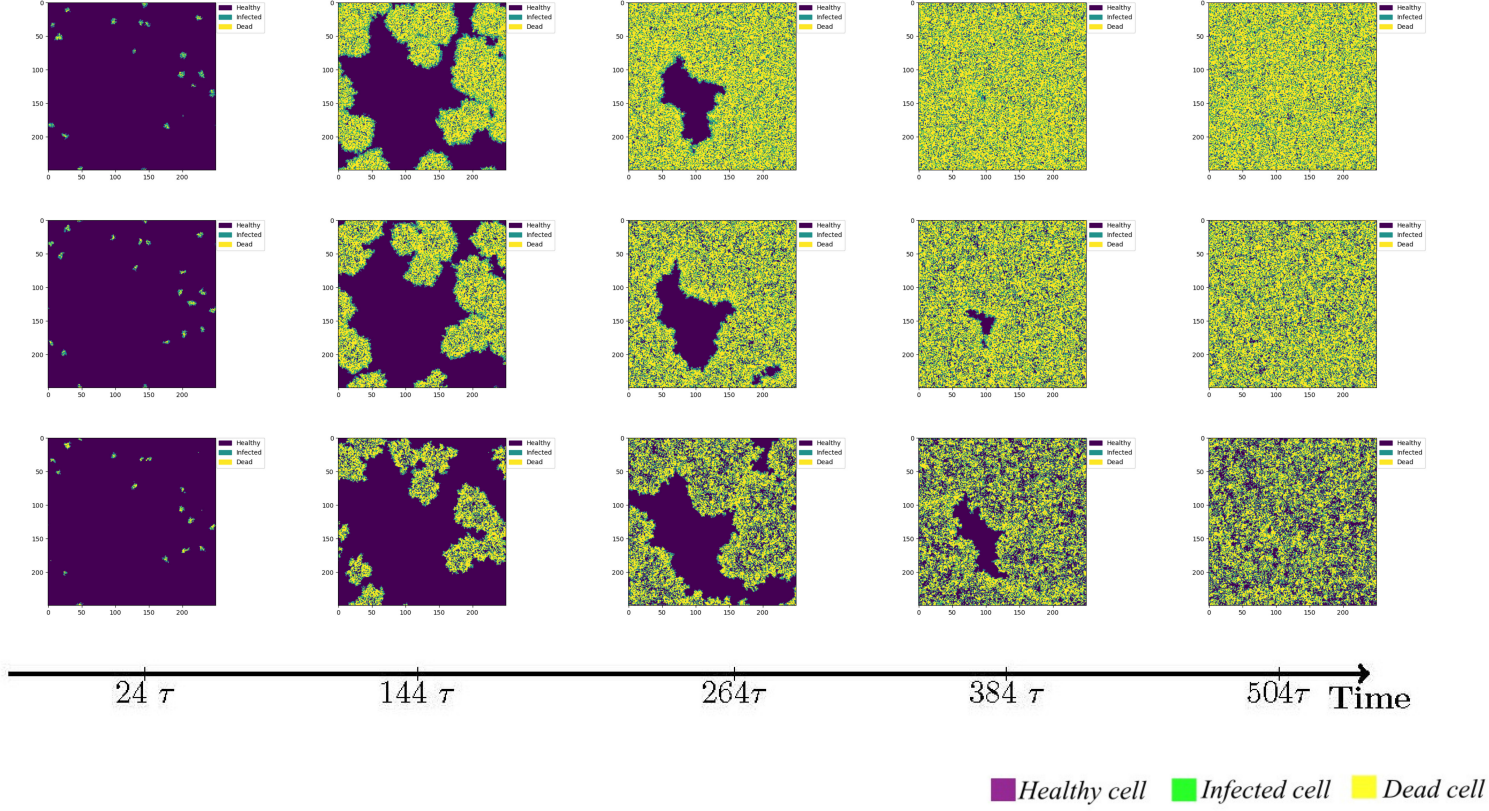
The status of cellular infection under different immune conditions of fixed immune points (Row 1), random walk (Row 2), and directional walk (Row 3).

However, it is important to acknowledge that immune cells are capable of migration, albeit constrained by intricate mechanisms. Although this study does not account for the physical barriers impeding immune cell movement, it is worth noting that immune cells possess the ability to wander in a random manner in all directions. Alternatively, a wandering mechanism can be implemented, wherein immune cells traverse a fixed step length of 1 cell units. Given that the area of virus-infected cells releases chemokines to attract additional immune cells, we further explore the scenario of immune cells exhibiting directional migration towards the infected region, thereby expanding their step length during wandering. The simulation results for these three immunization strategies are illustrated in **Figure 4**.

Under adaptive immunity, the presence of immune modulation significantly reduces the extent of cellular infection. Each row of images corresponds to a distinct form of immune regulation, namely fixed immune points (Row 1), random walk (Row 2), and directional walk (Row 3). Compared with results obtained without immunization (**Figure 3**, row 1), the three different forms of immunization exhibit varying degrees of effectiveness in containing the infection of cells. Although the inhibitory effect of fixed immune points and random walking immune mode is similar and weaker than that of directed walking immune mode, the rate of cell infection has slowed significantly, and the larger the number of health cells that have not been infected at the same time, as the immune intensity increases.

**Figure 5:**
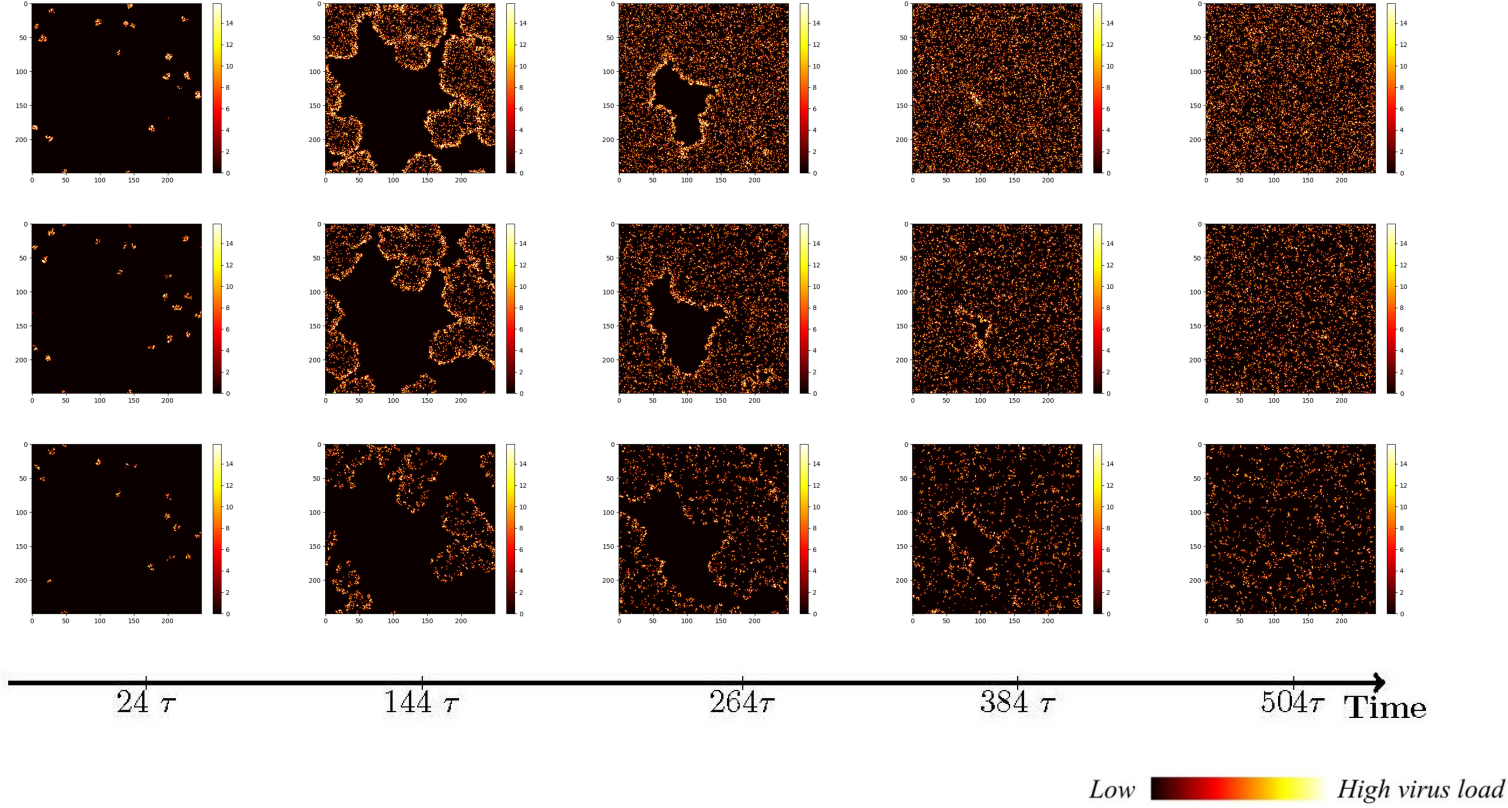
Viral spread under different immune regulation of fixed immune points (Row 1), random walk (Row 2), and directional walk (Row 3).

**Figure 6:**
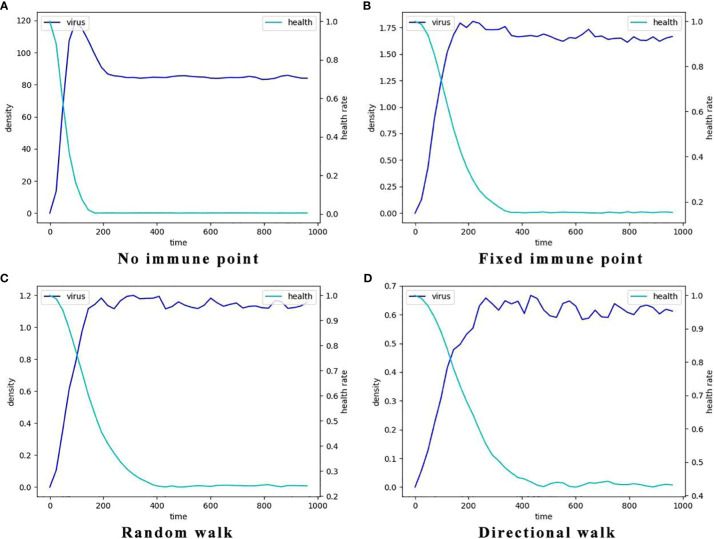
The typcial dynamics of virus density and the decay of the percentage of healthy cells in the four different immune cell movement modes. Here the light blue lines represent the dynamics of mean virus density which is indicated by the left y-axis and the dark blue lines represent the dynamics of the percentages of the healthy cells which is indicated by the right y-axis. **(A)** The dynamics without immune cells, i.e., the systems consists of only virus and epithelial cells. **(B)** The dynamics with static immune cells where the immune cells do not move (immune movement mode 1). **(C)** The dynamics with randomly moving immune cells (immune movement mode 2). **(D)** The dynamics with directly moving immune cells (immune movement mode 3).

We conducted an investigation into the influence of different forms of immune regulation on the process of virus spread and the occurrence of peak nodes. The findings are presented in the three-row plots depicted in **Figure 5**. As the intensity of immune cell suppression increases, notable changes in the dynamics of viral spread and peak nodes are observed. Specifically, the time point at which the peak of the virus is located moves backward, and the virus shows a continuous growth trend over a short period of time. As can be seen from the first two rows in **Figure 5**, the rate of virus spreading under the effect of the directed wandering immunity mode is weaker than that of the first two immunity modes, and a more obvious point is that the overall brightness of the third row of the picture is much lower, which suggests that the amount of viral proliferation has been significantly suppressed. These observations reveal the important role of immunomodulation in controlling virus transmission. Suppression of viral replication by the immune system leads to a shorter time to peak viral density followed by a gradual decline. These findings help us to provide a node for delaying large-scale viral outbreaks and reducing the extent of outbreaks to alleviate the pressure on healthcare resources. To provide a comprehensive understanding of the impact of the disease on human health and the dynamic changes in viral content within the body, a quantitative analysis was conducted. The results are depicted in **Figure 6**, it becomes evident that as the strength of the immune response intensifies, the proportion of healthy cells that become damaged gradually decreases, the proportion of healthy cells surviving increased from 20% to 40%.

Additionally, the node at which viral density peaks in the no-immunization mode (**Figure 6A**) is earlier than in the other three immunization modes. And in terms of peak viral density, viruses in the no-immunization mode showed a noticeable decrease in viral density after mass infection of healthy cells due to fewer host cells to parasitize. In contrast, viral densities in the other three immunization scenarios were maintained at relatively stable levels after reaching their peaks, and viral densities are at low levels.The most obvious of these is that the level of virus after peaking in **Figure 6D** is only about 0.6, a few hundredths of the level of the same variable in the no-immunization state. This suggests that the immune response does slow the rate of viral proliferation, delaying the point at which it reaches its peak and reducing the amount of virus infecting the human body.

### 3.3 Parameters regression analysis

In order to compare the parameter sensitivity of the model under different immunization modes, we performed Lasso regression analysis on all the parameters involved in the model and finalized the key parameters and their values under the three forms of immune site fixation, immune site random wandering and immune directed wandering. We introduce the following cost function, and key parameters affecting the rate of cell infection were identified by parameter fitting.


(7) $$Loss(w)=\sum_{i=1}^{N}{\left(y_{i}-w^{T}x_{i}\right)}^{2}+\lambda \|w{\|}_{1}$$

Among them, *x_i_* denotes the vector consisting of all parameters, including simulated and initial condition values, w denotes the weight of the parameter, and *y_i_* corresponds to the inverse of the cell infection time for different parameter values. We consider the *L* ^1^-paradigm of a w-vector with a penalty coefficient *λ* as the penalty term, which makes the weights of the less influential parameters 0. We need to find the value of w that minimizes the loss function.


(8) w=arɡ minw(∑i=1N(yi−wTxi)2+λ‖w‖1)

The parameter *x_i_* and its corresponding *y_i_* were entered randomly, and a large number of simulations yielded parameter sensitivities in different immunization modes.

**Figure 7:**
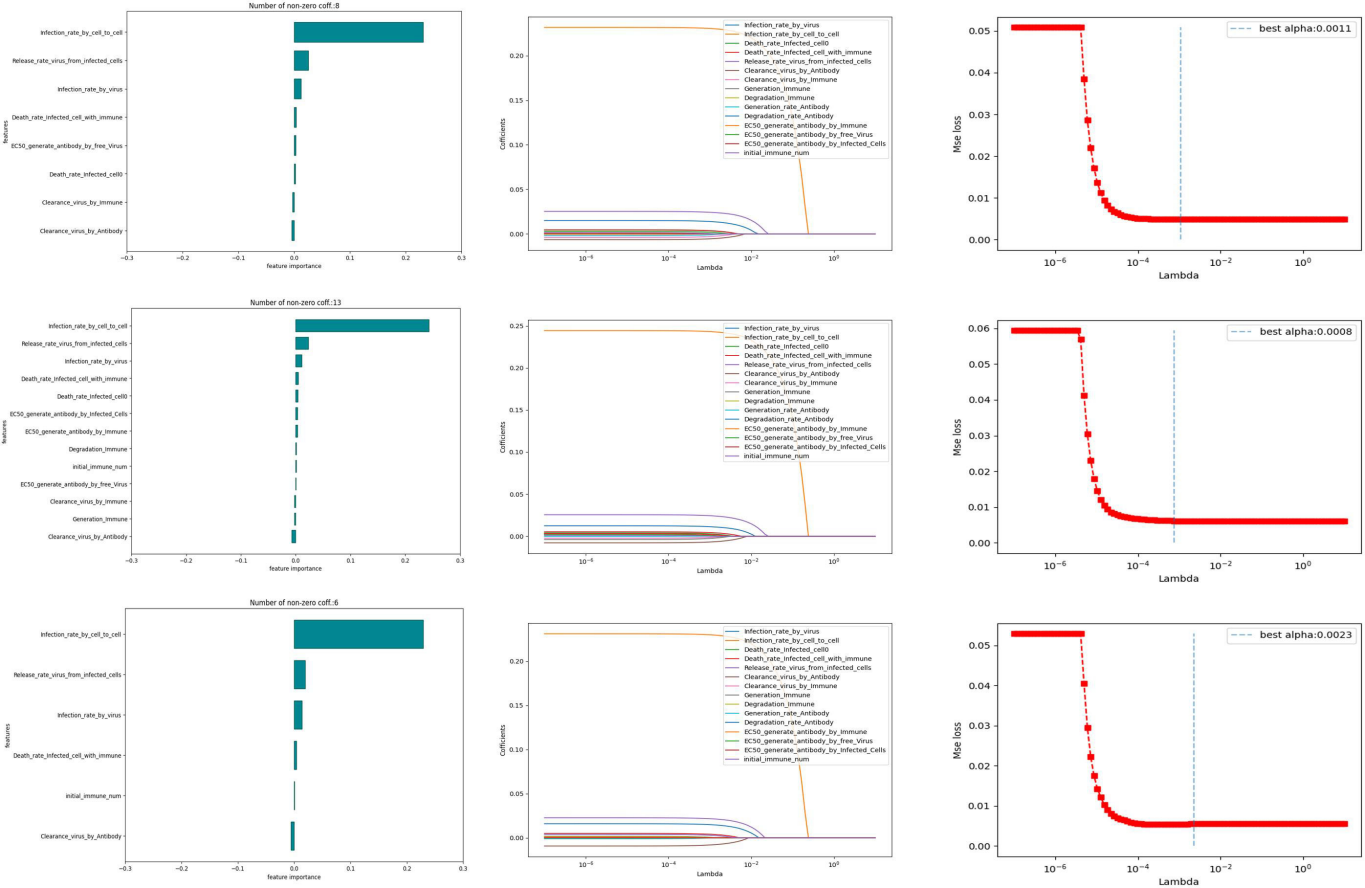
Potential sensitivity of model parameters to experiments.

We conduct 10,000 randomized computational trials and then employ the LASSO method to select the most important parameters. Different Lasso regression fits were performed for different models. The values of the parameters for the independent variables were taken from **Table 2**, and the dependent variable was the infection rate (the reciprocal of the time taken for healthy cells to reach 50%). From **Figure 7**, it can be learned that the number of non-zero parameters is different for different models. the non-zero parameter of the Fixed point model is 8, the non-zero parameter of the random walk is 13, and the non-zero parameter of the directional walk is 6. In addition, it can be learned that the data distribution of the weight coefficients of the three models is almost the same, and it is worth noting that the parameter Infection rate in cell-to-cell transmission is a crucial factor for all three models.

In order to further investigate the influence weights of the key parameters in the different models mentioned above, we conducted a regression analysis using OSL (Ordinary Least Square). In the results of the regression analysis, the experiment selects the top three ranked significant influences for different immunization scenarios. The **Table 3** shows the specific influencing factors and their weighting magnitude in OSL.

**Table 3:** Weights of parameters.

Immune models	Parameters	Weights
Fixed point	Infection rate in cell-to-cell transmission	0.1011
	Natural death rate of infected cell	−0.0427
	Initial virus density	−0.0397
Random walk	Infection rate in cell-to-cell transmission	0.1202
	Generation rate of immune cell	−0.0271
	Degradation rate of antibody	−0.0205
Directional walk	Infection rate in cell-to-cell transmission	0.1106
	Initial number of immune cell	0.0330
	Infection rate induced by virus	−0.0234

The first three influences with larger weights for the different models are as follows. As a whole, infection rate in cell-to-cell transmission is crucial for any immunization model. Note that. In the fixed point model, the second most important influence is natural death rate of infected cell, where a greater natural mortality rate of infected cells leads to slower spread. In the random walk model, the second most important factor is generation rate of immune cell. this is reasonable because, unlike in fixed point model, immune cells can travel, and thus the greater the probability of generating an immune cell, the more likely it is that it will cover more cellular tissue. In the directional walk model, Initial immune number has a larger effect on the model and a smaller effect on the two outer two models. This suggests that when there are more immune cells, a large number of immune cells move in the direction of more viruses and are better able to inhibit the spread of viruses.

### 3.4 The time for disease onset depends on several parameters

The progression of infection can be assessed by examining the changes in cell states at various spatial and temporal levels following viral infection in humans. As an important basis for judging the progression of the disease, the time point of onset is affected by many factors. Therefore, based on the above parametric regression analysis, this section will explore the influence of key influencing factors in the model on the time of onset (*T_onset_*) in different immune modes.

**Figure 8:**
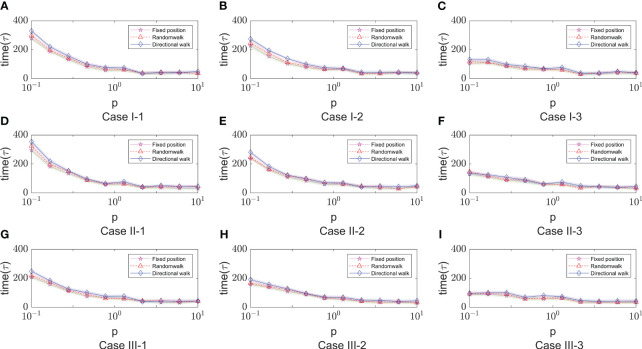
The time of onset (*T_onset_*) in different immune modes varies with the rate of cell-to-cell transimission rates. $p$ represents the values of cell-to-cell transmission rates. The three lines represent the dependence of the time of onset of disease on the parameter of the cell-to-cell transimission rates. Each row of figures represent a immune cell movement mode and each column of figures represent a typical dependence of the *T_onset_*. **(A–C)** are the cases of immune movement mode 1 which means that the immune cells are assumed to be static without movement. **(D–F)** represent immune movement mode 2 which means that the immune cells are moving randomly. And **(G–I)** represent the cases when the immune cells can move towards the direction of higher concentration of virus. While, for each column, **(A, D, G)** represent a longer onset time of disease indicating slow processes of infection; **(B, E, H)** represent a mediate rate of infection; and **(C, F, I)** represent fast processes of infection so the onset time is shorter than the other two groups.

After a large number of random simulations, we selected 9 sets of parameter values to adapt to different immune modes as shown in **Table 1**. Based on the content of the parameter impact weights in the previous section, we choose the impact factor (Infection rate in cell-to-cell transmission) that are important in any immune model to study and keep the remaining parameters as the analogical values of the corresponding Case. For the definition of morbidity, we assume that when the infected area reaches 50%, the infected person develops symptoms. The results of 9 sets of parameters are shown in **Figure 8**. There are three color bands in each sub-image, representing three immunization methods: Fixed point (Green), Random walk (Red) and Directional walk (Blue). The variable (Infection rate in cell-to-cell transmission) ranges from 0.1 to 10. Since at each parameter node, we performed multiple simulations, the broad bands of color bands actually represent the range of onset time fluctuations. For a more obvious comparison of the differences between the three immunization modalities, we split the representation into three mean lines. Consistent with previous simulations, the rate of virus growth was slowed and *T_onset_* delayed with increased immunosuppression. This is shown in **Figure 8**, where the color band indicating the directed wandering immunity pattern is higher than the other two in almost every Case. However, the differences between immune site fixation and random wandering are not significant. When the cell-to-cell transmission rate is large enough, the virus is able to rapidly infect a large number of healthy cells and reach the onset node in a short period of time. The three color bands gradually overlap, and differences in immune patterns no longer have a significant effect on the onset node. In addition, **Figure 8** (Case I-3, Case II-3, Case III-3) shows that the range of fluctuations of the onset node is significantly larger in the three immunization modes than in the other Cases of parameters. In these three sets of experiments, the average onset node of the different immunization modes was around 160 when cell-to-cell transmission was small, but as cell-to-cell transmission increased, the downward trend in *T_onset_* was slow and progressively greater than in the other six Cases of cases. This implies that there are other parameters that influence the course of onset. For this purpose, we further analyze the key parameters covered in the previous section.

**Figure 9:**
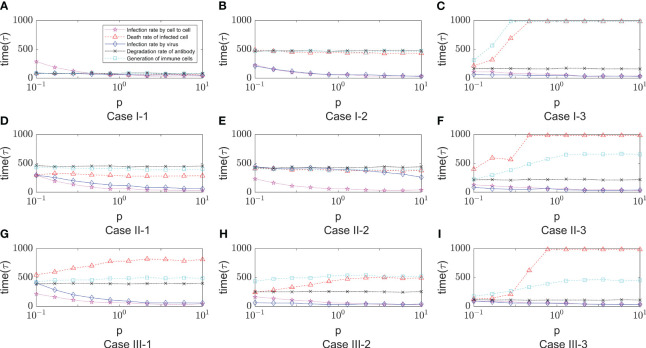
The dependence of the onset time of disease (Tonset) on five parameters, that is, the infection rate induced by cell- to-cell (x_6_), the death rate of infected cell (x_9_), the degradation rate of antibody (x_19_) , the infection rate induced by virus (x_5_) and the generation rate of immune cell (x_14_). The meaning of the different lines are shown in the legend of figure A. The x-axis represents the relative values of five parameters to the default values shown in **Table 1**. The five lines represent the dependence of the time of onset of disease on the five parameters. Each row of figures represents a immune cell movement mode and each column of figures represents a typical dynamics as those in **Figure 8**. **(A–C)** are the cases of immune movement mode 1 where the immune cells are assumed to be static without movement. **(D–F)** represent immune movement mode 2 where the immune cells are moving randomly. **(G–I)** represent the cases where the immune cells move towards the direction of higher concentration of virus with higher probability than other directions. Because the five parameters play a promoting or inhibitory role on the regulation of the onset time, for each column, **(A, D, G)** represent a longer or shorter onset time of disease indicating fast or slow processes of infection under the regulation of different parameters, and the same goes for the third column **(C, F, I)**, while the column **(B, E, H)** represent a mediate rate of infection.

As shown in **Figure 9**, it shows the effect of some non-overlapping parameters on the time to onset of disease in infected individuals in a parametric regression analysis. We consider the initial number of immune cells as an assumable constant. However, this study is more concerned with the influence of various biochemical reactions on the disease process during the interactions of the virus-induced immune response, so this factor has been eliminated and only the role of the remaining five factors has been explored. Most intuitively, the degradation rate of antibodies has essentially no effect on the rate of virus infection. As the infection rate induced by virus increases, the process of infection becomes faster, which is also very realistic. For cell-to-cell transmission, healthy cells become more susceptible to be infected because the infection rate induced by cell-to-cell shorten the time to onset of disease. This is shown in **Figure 9** (Case I-1, Case I-2, Case II-1 , Case II-2, Case III-1), the purple lines show that the onset time is decreasing with the infection rate induced by the cell-to-cell transmission while others do not show significant changes. By comparing the parameters, we find that *ρ*_0_ (Release rate of virus from infected cell) is smaller in the five Cases according to **Table 1**. This is consistent with the fact that increasing the rate of infection mediated by cell-to-cell promotes the process of onset when virus generate slowly. Usually, the rate of proliferation of immune cells increases the longer it takes for healthy cells to reach 50%. It can be seen that the blue curve in **Figure 9** (Case I-3, Case II-3, Case III-3) showed a significant upward trend, with little fluctuation in onset time in the other Cases. This is also due to the viral release rate, i.e., the small viral release rate leads to the low viral density and the time of onset can not fluctuate significantly even if changes in the number of immune cells. **Figure 9** (Case I-3, Case II-3, Case II-3) also shows that after the generation rate the immune cell reaches a certain level, its effect ceases to change. As for the death rate of infected cells, the effect on the rate of infection is more complex. The results shown in **Figures 9C, D, F** show similar patterns, that is, with the increasing in death rate of infected cells induced by the immune system, the onset times *T_onset_* keep unchanged for when the death rate (p) is greater than a certain threshold. This pattern is also found for the dependence of the onset time on the generation rate of the immune cells (the blue line in **Figure 9C**). This is due to the finite simulation period so they indicate that the onset time is longer than the simulation period rather than that the actual onset time kept unchanged with the increasing in parameters.

## 4 Discussion

This paper focuses on virus transmission targeting cell-to-cell in alveolar epithelial tissue. A hybrid partial differential equation is established. The virus propagation process is de-modeled by metacellular automata. In the establishment of the model, the experiment considers the epithelial tissue module, virus module, immune module and antibody module, and a series of parameters are set to simulate the experiment. Firstly, the experiment set up the modes of no immunization, immune point fixed, immune point random wandering and immune point directed wandering according to the presence or absence of immune response. From the results, the immunization effect of random wandering is almost the same as that of the fixed case, while the immunization effect of the immune site with directional wandering has a significant advantage. Next, we also compared the cellular infection under different diffusion coefficients. When the diffusion coefficient is larger, the virus is more evenly distributed and the peak value is lower. In particular, when the diffusion coefficient is large enough, the time for the virus to reach the peak is significantly delayed.

In addition, we experimentally randomly generated 10,000 sets of parameters for different immune models, defined healthy cells reaching 50% as the onset condition, and used the rate of onset as an indicator to derive the three main parameters that have the greatest influence on the infection rate in each immune model based on Lasso analysis and OSL regression analysis. The results showed that the infection rate induced by cell to cell had the greatest weight in each immunization model. Then, we selected nine sets of parameter values from the random simulation to carry out further analysis of the important parameters obtained from the regression analysis. It was experimentally demonstrated that the cell-to-cell transmission rate is large enough, the virus is able to rapidly infect a large number of healthy cells and reach the onset node in a short period of time, differences in immunization patterns had little effect. Whereas, in the general scenario, the directed wandering immunization mode suppresses the onset node better than the other two immunization modes. We found that the decay rate of antibodies had a small effect on the time of onset. The cell-to-cell transmission rate has a significant effect when the rate of virus release is low. The mortality rate of infected cells is relatively sensitive, and when the release rate of virus is high and the mortality rate of infected is also high, there will be a situation of reaching an infinite delay in the onset of disease, etc.

Regarding potential future improvements and applications of our model, we propose three main points. Firstly, the immune response is a complex and dynamic process involving various molecular, cellular, and regulatory pathways. Future work could incorporate these micro-level forms of immunity into cellular automata simulations to quantify the impact of the immune system on the spread of the novel coronavirus. Secondly, while there are numerous computer simulations of virus spread in populations, there are relatively fewer simulations focusing on virus spread within human individuals. Our study only considered the directional travel mechanism of immune cells and major immune cell types, without accounting for factors such as travel speed and changes in the number of immune cells. Further investigations could explore the effects of these factors on virus spread within the human body. Additionally, the assessment of morbidity could be improved by considering alternative perspectives. Finally, for the section on the analysis of parameters affecting onset time, the article has yet to explore under what circumstances the regulation of certain parameters would be ineffective or less volatile or more sensitive to parameter changes.

## Data availability statement

The raw data supporting the conclusions of this article will be made available by the authors, without undue reservation.

## Author contributions

YC: Conceptualization, Methodology, Writing – original draft. ZZ: Data curation, Software, Visualization, Writing – original draft. CZ: Formal Analysis, Project administration, Supervision, Writing – review & editing, Funding acquisition.
